# 

**Published:** 1869-10

**Authors:** 


Three Dollars a year. Single Copies, 30 Cts.

VOLUME XXVI. *OCTOBER*, 1869. NUMBER XIX.

THE

### Chicago Medical Journal,

A MONTHLY RECORD OF

*Medicine, Surgery & Collateral Sciences.*

J. ADAMS ALLEN, M.D., LL.D., WALTER HAY, M.D.,
EDITORS.

CHICAGO:
W. B. KEEN & COOKE, PUBLISHERS,
113 & 115 STATE STREET.

To whom communications for the Editors and books for review must be addressed.
The postage on this Journal is 24 cents a year—payable quarterly in advance.

Church, Goodman & Donnelley Printers

